# Children’s and adolescents’ views of health and mental health concepts - A qualitative group interview study

**DOI:** 10.1186/s12889-024-20042-6

**Published:** 2024-09-16

**Authors:** Sven Hassler, Siri Jakobsson Støre, Louise Persson, Linda Beckman

**Affiliations:** 1 Department of Health Sciences, Karlstad University, Karlstad, Sweden; 2 School of Behavioural, Social and Legal Sciences, Örebro University, Örebro, Sweden; 3 Department of Social and Psychological Studies, Karlstad University, Karlstad, Sweden; 4 Department of Health Services Research, Management and Policy, University of Florida, Gainesville, US

**Keywords:** Adolescent, Children, Content analysis, Group interviews, Health, Health literacy, Health promotion, Mental health

## Abstract

**Background:**

Definitions and perceptions of health and mental health have not remained static over time. This is also true for statistics over Swedish children’s and adolescents’ health and mental health status. The majority of Swedish school-aged children and adolescents report good physical health and good life satisfaction. However, there are some warning signs when it comes to children’s and adolescents’ health and mental health status, for instance, an increased overweight or obesity in children and adolescents, as well as a higher proportion reporting psychological problems and stress. There is also a need for knowing more about the younger population’s voices in this matter. The aim was therefore to explore children’s and adolescent’s conceptualizations and perceptions of health in general, and mental health in particular.

**Methods:**

Open semistructured group interviews with 44 Swedish children and adolescents (10–14 years old) recruited from four schools were conducted. The interviews were conducted between April 2022 and January 2023. Data were analyzed with qualitative content analysis.

**Results:**

Children’s and adolescents’ conceptualizations of health included aspects of both the body and the mind, with a focus on the latter. Mental health was expressed as a state of being, illustrated by various lived experiences of emotions, moods, and thoughts. The social world was ever present in their understanding of health, e.g., through the lenses of social and gender norms.

**Conclusions:**

This study revealed children’s and adolescents’ recognition of health terms and their ability to observe nuances between mental health problems and everyday struggles. The participants discussed mental health problems to a greater extent than positive mental health. An implication of this study is the highlighted need to focus more on mental health promotion in future preventive programs. These findings might potentially influence how school staff and student health teams communicate with children and adolescents about these concepts.

**Supplementary Information:**

The online version contains supplementary material available at 10.1186/s12889-024-20042-6.

## Background

The majority of school-aged children and adolescents in Sweden have good physical health, and report good life satisfaction. However, around 25% of the 6–10 years old school children are overweight or obese, and far from all are physically active enough [1, 2]. Many children and adolescents also experience stress and report psychological problems, painting a complex and elusive picture of their health and well-being [3]. Common indicators of mental health problems among school-aged children and adolescents include anxiety, worry, sleeping problems, and low mood. A high percentage of children also express feeling stressed by schoolwork, with 15-year-old girls reporting the highest percentage (78%) compared with boys of the same age (51%) [3].

Further, throughout the 2000s, visits to Child and Adolescent Psychiatry (CAMHS) in Sweden dramatically increased [4]. However, whether this reflects an actual increase in illness or a shift in demand, diagnostic criteria, and school dynamics remains inconclusive. As discussed in Lindholm and Wickström [5] and Wickström and Kvist Lindholm [6], professionals working with children and adolescents note that “crises” once considered normal aspects of maturation and development in children and adolescents risk being increasingly categorized as mental illness [7]. Life crises and everyday behavior may become medicalized, leading more individuals to seek assistance from CAMHS for issues that were previously addressed within family or social circles, such as relationship breakups (8–9). Moreover, a growing number of children and adolescents present with challenging and complex needs, indicating a diverse demand for assistance [4]. Thus, a medicalization of everyday challenges seems to have expanded the scope of health and mental health. This development reflects a changing society and a new coping mechanism for emerging realities.

The concept of health and mental health has been discussed in the literature for a long time. WHO have defined health as: “a state of complete physical, mental and social well-being and not merely the absence of disease or infirmity” [10], and have been criticized for, among other things, being too idealistic, referring to the statement of a complete health [11]. Children and adolescents’ perceptions of health as a concept have also been scrutinized. Research shows that children and adolescents, instead of defining health in terms of absence of chronic sickness, tend to use psychosocial health and health-related behaviors (such as sports activity, (12–13), being happy, feeling good [14], diet/healthy food, fruits and vegetables not smoking and sleeping) [15] as criteria.

Definitions and understandings of mental health - a relatively new concept - have not remained static. A process of reconceptualizing has led to uncertainty in two ends. In one end the functional understanding of the individual, public lay members and in the other the pragmatic use of the concept by policy workers and academia (16–17). Manwell et al. [17] suggest that the widespread use of the term ‘mental health’ serves as a euphemism for ‘mental illness’ and that the lack of agreement on the subcomponents and factors encompassed by the concept underscores the need for distinct definitions for individual and societal understanding.

As mental health often is used in general conversation but given different meanings in different contexts, a uniform use of key concepts related to mental health has been proposed [18]. It is notable that positive mental health is represented solely by mental well-being while negative mental health has a multitude of expressions and representations such as mental illness, mental problems, psychiatric conditions, neuropsychiatric impairments and mental illnesses and syndromes [18].

A mapping by Manwell et al. [17] of how mental health experts determine core concepts illustrates how different empirical discourses shape mental health definitions. There is the definition proposed in the spirit of health promotion, emphasizing the positive aspects of the concept. Mental health is seen as the capacity for individuals to feel, think, and act in ways that enhance their ability to enjoy life and cope with the challenges, respecting culture, equity, social justice, interconnections, and personal dignity [19]. The World Health Organization’s (WHO) definition echoes similar positive dimensions: “a state of mental well-being that enables people to cope with the stresses of life, realize their abilities, learn well and work well, and contribute to their community” [20]. The criticism of such broad definitions highlights their contributions to the medicalization of society by viewing everyday problems within the scope of mental health [16]. An argument supported by Graham [9] who finds that the discourse of wellbeing is underpinned by an individual disease model and as such, is inherently medical in concept [9].

A previous scoping review [21] on children and youth’s perceptions of mental health revealed some uncertainty in the understanding of the concept. A shared mental health language would help children clarify distinctions between everyday challenges and issues requiring specialist attention. According to Beckman et al. [21], highlighting the positive aspects of mental health can increase the understanding that mental health can be improved. Similar conclusions are drawn by Hermann et al. [22], who emphasize the importance of clear terminology in mental health discussions. They further demonstrated that older adolescents (15–18 years) discuss mental health in holistic terms, indicating their understanding of its complexity despite the use of simplified language.

The various discourses around the concepts of health and mental health raise questions about whether we can expect children and adolescents to understand and communicate about them. Empirically investigating perceptions of health among children and adolescents needs to consider researchers’ presumptions of these concepts. As a response to the biomedical approach, Antonovsky [23] introduced the salutogenic approach to health, which views health as more than just the absence of risk factors. It focuses on human strengths, viewing health as a continuum rather than a dichotomy. Mental health in a salutogenic perspective refers to the level of health and resources present that can be recognized, utilized, and nurtured. Also acknowledge tension as a normal and necessary feeling for coping and therefore potentially health-promoting [24]. Consequently, there is a pressing need for more studies involving children and adolescents’ voices to gather their perspectives on the health concept and mental health.

## Methods

Understanding how health and mental health are perceived and communicated among children and adolescents is crucial in planning interventions and strategies for health and mental health care and support. Therefore, this study aimed to explore children’s and adolescent’s conceptualizations and perceptions of health in general, and mental health in particular. With the explorative approach of the study, we have chosen to stay rather free from newly proposed conceptual frameworks of mental health as our ambition is to empirically add information and shape those frameworks rather than to apply them. Our broad and open approach motivated us to even include health in this conceptual mapping.

We opted for group interviews to gather in-depth information from the participants, foster comfort within their peer groups and peer support [25], facilitate the exchange of experiences, and enable them to comment on each other’s viewpoints. The interview guide was not followed strictly. According to Leeson [26], letting the children guide the conversation will help the researchers see what is important to them.

### Data and participants

This study involved 44 participants (26 girls and 18 boys) aged 10–14 years from four different schools in Värmland County, Sweden. This age range was chosen because it comprises grades 4 to 8 in Swedish schools, and because children of that age could be considered old enough to discuss these questions and understand what they comply with. Eight mixed-gender group interviews were conducted from April 2022 to January 2023 (see Table 1 for the gender and grade distributions). The interviews took place at the participants’ respective schools.

**Table 1:** Number of participants by groups and grade in each school area respectively

**North School**	**9**	**5**	**4**
Group 1			
Grade 8	4	2	2
Group 2			
Grade 8	5	3	2
**East School**	**17**	**10**	**7**
Group 1			
Grade 6	6	5	1
Group 2			
Grade 5	6	1	5
Group 3			
Grade 4	5	4	1
**South School**	**10**	**7**	**3**
Group 1			
Grade 5	6	4	2
Group 2			
Grade 4	2	2	0
Grade 5	2	1	1
**West School**	**8**	**4**	**4**
Group 1			
Grade 8	8	4	4

### Procedure and interview guide

The study received approval from the Swedish Ethical Review Authority (No: 2021-06168-01). Central Student Health Services (SHS) assisted in selecting schools and recruiting participants from grades 4–8 at four different schools. Subsequently, the schools were contacted and provided with documents and project information. Participants were recruited from classes through school personnel (principals, school nurses, and school social workers). No specific selection criteria were outlined, except that the participants were required to have their parents’ signed consent at the time of the interview. Children and adolescents interested in participating in a group interview were eligible for the study. Some schools opted to inform the entire class about participation, whereas in other cases, teachers approached specific children. The participating children received both oral and written information about the study, emphasizing its voluntary nature, their right to terminate participation at any time, and the assurance that their responses would remain confidential. Written and oral informed consent was obtained simultaneously with the interviews, which took place approximately two weeks after the first information session. The group interviews were conducted using open-to-semistructured interview techniques, with an interview guide developed on the basis of previous literature, such as Beckman et al. [21], which focused on amplifying children’s voices on health matters. Slightly different questions were tailored for different age groups so the questions would fit their ages and maturation (Appendix A).

The interviews were conducted by all the authors, with two authors present for each interview, alternating between being the roles of moderator and co-moderator. The moderator posed the questions and followed up with probes such as “Can you elaborate on what you just said?” and “Can you provide an example?”. Before concluding each group interview, participants were invited to share any additional thoughts or highlight any overlooked topics. The interviews were audio recorded digitally and the interviews lasted between 30 and 59 min. The participants were not requested to review the transcripts before analysis because of potential embarrassment or confusion in distinguishing their statements from others in the group [27]. As the schools were chosen by the school health team manager, the sample could be considered a convenience sample. The socioeconomic status of the schools was assessed as middle to high on the basis of grade points and average income for the district where the school is located.

### Data analysis

Data analysis was conducted via the qualitative content analysis described by Graneheim and Lundman [28] and discussed by Graneheim, Lindgren and Lundman [29]. In the first step, each group interview was transcribed verbatim, and all the authors read the transcription of each group interview numerous times. Descriptions of health and mental health constituted the unit of analysis. Next, the meaning-carrying units were condensed and abstracted into codes. These codes were then compared and sorted into categories. As the analysis progressed, the categories and subcategories were further clarified and adjusted, resulting in the creation of three categories (Table 2). The initial coding of the transcripts were generated by SH, SJS, and LB, each coding different interviews. The data were then collectively examined to construct categories. Comparisons were made with the context in each step to verify the empirical basis of the data. Tentative codes and categories were discussed by all the authors and revised until consensus was reached. In cases of disagreements, we revisited the meaning-carrying unit to ensure the fit with the category or reconsidered the preliminary coding. Reflection and discussion led to agreement on sorting the codes. By including quotations from the transcribed text, similarities within and differences between categories were illustrated [28]. The quotations were translated from Swedish to English with the help of a native English speaker. The quotations cited in the results are labeled on the basis of school and grade. The current study is reported following the Consolidated Criteria for Reporting Qualitative Research (COREQ) checklist [29]. In conducting qualitative studies, it is important to acknowledge an awareness of the researchers’s pre-understanding to minimize its influence on the results and analysis. However, eliminating it entirely is neither possible nor desirable. The challenge is to make clear whose voice is heard: the participants’ voice or the researchers’ interpretation [29]. According to Alvesson and Sandberg [30], preunderstanding can broaden the empirical base, generate ideas, formulate research questions, and evaluate the relevance and novelty of knowledge. The authors of this article have diverse backgrounds in social science, public health sciences, and psychology, contributing to - and constraining - the understanding and interpretation of the results.

### Trustworthiness

To enhance trustworthiness, each step of the study included discussions concerning credibility, dependability, and transferability [28]. Credibility was bolstered by including a variety of participants from various schools, striving for diversity in age and gender. Our research team also included members with different backgrounds, allowing for a multifaceted examination of the data. We believe that the choice of group interviews further enhanced the credibility since children may feel a sense of imbalance in power dynamics when meeting an unknown person alone and discussing health and mental health [31]. Additionally, we developed an interview guide ensuring that the same questions were addressed in all interviews, further bolstering credibility. To reinforce credibility, we included representative quotations from the interviews, and thoroughly discussed the codes and preliminary categories. The research team included experienced interviewers, as well as a licensed Psychologist specializing in clinical child and adolescent psychology (PsyD) (i.e., an expert on mental health), which have contributed to maintaining a safe environment, particularly if the discussion touched sensitive topics such as suicide [26]. Dependability refers to the stability of data over time [28]. Seven of the eight group interviews were conducted during late spring and after the summer holiday, with one conducted the following winter. We believe that the gap between the seventh and the eighth interviews likely did not affect the results. While new follow-up questions may have emerged during the later interviews, we did not review the transcripts beforehand. However, we do not consider this a factor contributing to inconsistencies in results but rather a source of greater variation in answers. The description of culture and context, characteristics, and selection process enables readers to assess the transferability of findings to other groups and contexts [28]. Owing to the nature of the sampling, which is partly adapted to school circumstances and the availability of time and willing teachers, transferability is open to discussion. Nonetheless, by selecting schools from different socioeconomic districts and including boys and girls across grades 4–8, we believe that the sample can be considered transferable within a cultural context comparable to that of Sweden.

## Results

The analysis resulted in three categories: (1) *Conceptualizations of health* (2), *Experience-based manifestations of being*, and (3) *Social norms*. Please see Table 2 for an overview of the categories and belonging subcatogories.

**Table 2:** Overview of the categories and subcategories

Categories	Conceptualizations of health	Experience-based manifestations of being	Social norms	
**Subcategories**	Health - body and mind	Health as a state of being	Fitting in and being affirmed	
	The meaning and value of the word	Negative mental health as an elusive emotion	Complexion and self-image	
	Overlapping concepts	Health affected by sources of worries	Social life in school and on social media platforms	
			Tell, show, ask	

### Conceptualizations of health

The first category included the subcategories *Health - body and mind; The meaning and value of the word* and *Overlapping concepts.* In conceptualizations of health, the participants gave their view of what health, mental health, and similar concepts meant to them.

#### Health - body and mind

When asked to describe what *health* is, the participants discussed diet and exercise – especially the younger ones. Good health was associated with the ability to eat nutritious food and essential vitamins, and to generally avoiding eating junk food. Staying “fit”, i.e., maintaining a stable weight was also considered an important aspect of good health. It was evident that some children’s conceptualization of health encompassed mental well-being, and that these children recognized an interconnectedness of the body and the mind:

These factors affect each other. Therefore, if you have positive thoughts and feel good mentally, it might be easier for you to be active and eat well. For some, it could also be that staying active and sleeping well may lead to better thoughts and such. (North School, grade 8b)

I would say that they are two completely separate things: one is about how your body feels, and the other is about how you feel mentally. (East School, grade 5)

Although some younger students in Grade 4 were unsure of the meaning of mental health, it was described as “how you feel in your brain”, “that you are happy and energetic”, how you are treated by others, and how you enjoy your surroundings. Mental health was also associated with academic performance and peer relationships. However, understanding someone’s true emotional state was acknowledged as challenging:

For some people, you might think that they’re doing really well, but deep down, they may not always be feeling great. Mental health can encompass many things because much is happening under the surface. (South School, grade 5)

One pupil in Grade 6 described mental illness as unwell in the mind, emphasizing that it involves prolonged feelings of sadness rather than transient emotions. There was a perception that mental health was more important than physical health. Taking care of one’s body through exercise was seen to positively impact mental well-being, and that the two are intricately linked. A fifth-grader illustrated this by emphasizing that you need to work harder to get rid of a state of feeling mentally unwell than being physically sick:

I think there’s more weight in regards to the mental aspect because when you’re feeling mentally unwell, you have to put in extra effort to overcome it. Whereas, if you’re physically unwell, it often tends to pass after a while, such as when you’re sick. (East School, grade 5)

#### The meaning and value of the word

A call for greater caution was evident in how the concepts related to mental health should be used. The participants expressed concern that mental illness was often trivialized through humor, risking its significance being undermined. With respect to mental health concepts, the participants stated: “One must know what the word means”. One fifth grader provided an example of the non-sarcastically use of a loaded expression:

Sometimes people may joke about being depressed, like saying, “Oh, the game got canceled today; I’m so depressed now.“(East School, grade 5).

The more general expression “feel bad” was described as a collective term that could carry varying degrees of seriousness:

You don’t necessarily have to use it (feel bad) for mental problems; if you have a stomachache, you feel unwell. The weight of the term varies depending on the context in which it is used. (East School, grade 6)

The multiple views of what constitutes mental health indicate the complexity of mental health-related concepts, highlighting the challenges in their usage and understanding across different contexts.

#### Overlapping concepts

With respect to the concepts related to mental health, the children reported that their perceptions of these concepts frequently overlapped. Concepts such as sadness appeared to be perceived as easier for the children to cope with and worry was perceived as different yet interconnected. The participating children and adolescents disclosed that it was more challenging to feel worried than sad. One reason for this was, “When you are sad, you can cry, but when you are worried, you have to talk.” (East School, grade 6). Dealing with sadness could be perceived as “easier”. Worry, on the other hand, could be experienced as stressful, with stress being described, among other things, as a mixture of feeling sad and worried. Stress was characterized as a sensation that “took over,” but it was acknowledged that there could be both positive and negative forms of stress. Anxiety was seen as an extension of stress, where individuals tend to “overthink” various situations and events:

Anxiety is a combination of stress and feeling a bit down. Homework, negative social circles, exams, and things that weigh you down can lead to a point where you cannot handle it anymore, resulting in a panic attack or something similar. (North School, grade 8a)

Anxiety was described as a sudden and intense feeling that could cause difficulty in breathing. However, older children believed that anxiety was not a term commonly used and was considered “harsher”. Panic attacks were viewed as an escalation of anxiety: “Stress is more common; a panic attack is the peak of anxiety.” (North School grade 8a). During a panic attack, everything could seem to freeze, and there could be a sense of darkness for a while.

Yes, I believe there’s a difference between anxiety and panic attacks. It seems that anxiety is something that lasts for a longer period, possibly transitioning from stress to anxiety. Many different factors can trigger this anxiety. However, when it becomes a panic attack, I think it is more intense and happens closely together. That is what I think. (North School, grade 8a)

When asked to define depression, it was described as feeling unhappy, very low, lacking the motivation to take care of oneself, and struggling to get out of bed. It could involve overthinking, “worrying” and burying oneself. There could also be different versions of depression, where some deny it and go around smiling all day, but then when they get home, they dive into bed and are “completely destroyed”. The children said that everyone feels bad sometimes, but depression is being down for a longer period of time and that it was important to get help.

### Experience-based manifestations of being

In contrast to the perhaps more knowledge-driven conceptualizations of health, experienced-based manifestations of being represent answers relating to the inner self and one’s own experience of ill- and well-being. Experience-based manifestations of being include the subcategories *Health as a state of being; Negative mental health as an elusive emotion*,* and Health affected by sources of worries.*

#### Health as a state of being

Health is expressed by the participants as consisting of various expressions of emotions, moods, thoughts, and overall well-being. If conceptualizations of health represent explicit descriptions of concepts, health as a state of being represents hints of ideas originating from one’s own experiences, implicitly relating to health through aspects of being. The spectrum on which these expressions move stretches from concrete expressions regarding physical health and sedentary lifestyles to vague and elusive expressions of being. The former is more often a response to physical health. Feeling fit and alert, being physically active, and sleeping properly were seen as expressions of well-being, whereas inactivity represented mental illness. A fourth grader gives this example:

I think bad health is mostly when you sit all the time and do not do much. (South School, grade 4)

#### Negative mental health as an elusive emotion

There is an elusiveness that characterizes these perceptions, mostly related to the negative aspects of being or moods, where the participants seemingly do not quite grasp the word that correctly describes the feeling or state of being. An elusiveness in contrast to others, more clear illustrations of mental health such as being happy, in good spirits, or enjoying oneself. However, negative perceptions of mental health manifested as a form of ill-being, often more vaguely expressed as a certain emotion, thought, mood, or gut feeling. Something they carry with them, sometimes for longer periods, as a state of being of the inner self.

It kind of doesn’t feel good. (East School, grade 5)

I think it means psychologically, it’s inside, that is when you kind of do not feel good inside and that it is like, it is like that thing with stress, that you do not feel so good inside. (East School, grade 4)

#### Health affected by sources of worries

Worries are also perceived as something that triggers mental illness and stress and are expressed in different forms and distances from oneself. Worries about the war in Ukraine and Syria or worries that a family member will die or become hurt. However, worries about school and schoolwork were also quite extensively expressed. These worries as expressions of mental illness resemble what was also perceived as stress. When asked how they would describe stress, associations with thoughts were quite common, and they thought of something that made them stressed. Some describe it as overthinking or as a feeling that ends up in the stomach and creates anxiety. School education seems to be the source of much anxiety and stress. Stress about tests, grades, and not learning enough or as a general feeling of being chased and unable to make it. A feeling apparently tangible and overwhelming to the informants but seldom concretized or further described. A sixth grader emphasizes stress when it is too high.

One becomes overwhelmed, you know. You might think, for example, that you have many things to do, such as studying, exercising, and many other things, and it can become a bit too much. (East School, grade 6)

### Social norms

The social world was ever-present in children’ understanding of health, either implicitly or explicitly. Social norms included *Fitting in and being affirmed*,* Complexion and self-image*, *Social life in school and on social media platforms*,* and Tell*,* ask*,* show*. The children viewed fun activities with friends as having beneficial health effects because you do something enjoyable (and hence good) for yourself in a shared experience with close ones. However, social relationships are also associated with potential disadvantageous health consequences. “Being normal” was considered vital for health, and it was also evident that beauty standards and gender norms were related to mental health and showing emotions.

#### Fitting in and being affirmed

Being normal and fitting in were considered essential to health; it is important to look and act like everyone else. If you do not fit in, you are left out:

And it can also be that you feel like, ‘I do not fit in here; everyone else is so cool, and I just come in here suddenly like a little…’. Yeah, it is probably mostly feeling a bit excluded or left out. (South School, grade 5)

Mental health was, in this context, described as being affected by how people view you, what you do, and whether you are liked or not and feeling affirmed.

Yes, I can get sad sometimes, I can think about what people think of me, or you really want to be liked, and sometimes you might just feel that you think you’re really ugly or something and that, yes, you get very depressed. (South School, grade 6)

On the same note, being socially awkward or prone to overanalyzing social situations can be stressful and depressing. Conforming to the group may lead to a sense of belonging, but chasing social affirmation may be compromising your health, e.g., doing things you do not want to do through peer pressure:

You do things you do not want to, as others want it, and so you want to be with them. So instead of saying, ‘No, I don’t want to do this, can’t we do anything else?‘, you do it anyway, so you do not exactly take care of yourself. Additionally, you sit there feeling sad because you did something that made you feel sick or bad. (South School, grade 5)

#### Complexion and self-image

Mental health is also described with reference to how one feels about how one looks and appears in the eyes of others. There seems to be a definitive link between physical appearance and mental health, and it occurs in both ways. To desire something you do not have, a look or appearance, was one way of describing how mental health could be affected by complexions or one’s self-image. Trends in looks and body shapes are other examples of what could affect mental well-being.

So it could also be that you feel bad, for example, about your appearance or you feel that you are not enough, so then you think that you are mean even though you are not satisfied with yourself, and then it becomes psychological. (South School, grade 5)

The body trends reflected in social media were considered unhealthy, and these beauty standards mostly affected girls - and made them feel worse.

#### Social life in school and on social media platforms

Well-being and ill-being as a signifier of health were also expressed in social terms, such as how things are going at school and how one handles life. Well-being and ill-being become extensive in the descriptions of children and adolescents; they can be about not feeling as usual, feeling scared, or not being good enough. It was perceived as part of life and an everyday occurrence, appearing to many of the informants not only as a consequence of social situations and encounters in real life, but certainly also on social media platforms. The participants expressed concerns about the ongoing talk but also as incitement around trends and behaviors. The participants’ experiences of social media were described as both positive and negative in terms of being seen and being part of a community, but it also led to more comparisons of oneself to others and more negative feelings. However, at the same time, informants referred to social media and the cell phone as a place to take a break and escape from stress at school, thus facilitating well-being. The children described how they try to act like everyone else on social media. Social media sets standards for how one should look, which can lead to stress and depression:

Social media sort of raises the standard of how one should look and dress. For example, many people Photoshop and tinker with the pictures, whether they want to get a smaller waist or larger biceps or, yes, to make it look better and: This is how a body should look - even if it is not supposed to look like that. (North School, grade 8a)

Many have experienced stress or depression because they want to look like those who have been Photoshopped. Yes, so there are also many disadvantages. (North School, grade 8a)

The adolescents and children also spoke about the role that social media has in spreading so-called fake news and, by that, spreading anxiety.

#### Tell, show, ask

The children and adolescents perceived it to be more of a taboo for boys to cry than for girls. They expressed that boys often abstain from crying to avoid being perceived as weird or non-masculine, even though they emphasized that everyone cries sometimes and that it is necessary to cry occasionally. Girls were described as having more reasons to be sad because they hurt each other more:

I’m not a girl myself, but girls seem to get more hurt by their friends, so it feels like it is quite difficult to be, well if there are three of them, two might hang out together, it can be quite difficult to be the third wheel. However, we guys can be a group of several boys without, well, without any issues, and it works truly well. (North School, grade 8b)

The children attributed these gender differences to society, with one explaining that there’s just this feeling in his head that it is how things work. Additionally, there is an age difference to consider, where it is believed that it is acceptable for young children to cry, but teenagers need to maintain a cool image to a greater extent. Crying in front of others would make you feel weak.

The children emphasized responsibility for others and the importance of asking friends how they are. They value the support of others but noted that it is “not something they usually do in their spare time”. The participants also expressed a risk of rejection when one opens up about how one truly feels, and friends may also recoil when asked about their feelings. It’s also a matter of integrity; you do not have to share *everything*, and you cannot force another person to share how they are feeling:

It is difficult to share because you do not need to disclose everything that has happened. You only need to share if you’re truly worried about something, like 'This is what I’m most concerned about how they will react and what they will think’. In that case, they can skip that part and share everything they feel comfortable sharing. (South School, grade 4)

## Discussion

Analyzing the data of school children’s views on health and mental health revealed several important information about how they understand these concepts. The results are first discussed in relation to each of the inductive coding categories: *Conceptualizations of health*,* Experience-based manifestations of being*,* and Social norms*. After that, the findings are discussed in relation to implications for practice and future research.

### Conceptualizations of health – the children are well aware

When we inquired about health in general, there was a significant emphasis on eating well and doing physical activity, mirroring the findings of another Swedish study that interviewed children between the ages of 9–11 [32]. Our main findings reveal that health and mental health are not unfamiliar concepts to school-aged children and adolescents, although the youngest children in Grade 4 struggled with mental health, which is consistent with findings from a recent scoping review [21]. However, mental health was also included in these discussions, and the two concepts - health and mental health - were seen as interconnected, reflecting the relationship between the body and mind. Mental health was sometimes perceived to be even more crucial than physical health.

Mental health was described by the youngest participants as how “one feels in the brain”, which encompasses feelings such as mood, emotions, and overall well-being experienced in the mind. It includes feelings of happiness and energy, findings that are consistent with other qualitative studies with children and adolescents (22, 33–34). The children also included social relationships and surroundings in their descriptions of mental health, a finding echoed elsewhere (33, 35–36). Mental health was also described as when nothing goes against you, akin to the feeling of harmony, as reported by Landstedt et al. [37].

However, mental health is also intertwined with general health, which risks further conceptual ambiguity. Manwell et al. [17] proposed that the integration of mental and physical health can be defined by the level of autonomy (i.e., the capacity for control over one’s self), whereas the integration of mental and social health can be defined by a sense of ‘us’ (i.e.,the capacity for relating to others). A capacity that was observed in our material as the children associated mental health to peer relationships and how “you enjoy your surroundings”.

Depression, as part of mental health, was understood somewhat distinctly as characterized by not being happy and feeling very bad. The participants emphasized that everyone feels bad sometimes but that depression is feeling down for a longer period of time and that it is important to seek help. Similar findings were reported by Hermann et al. [22], where depression was viewed as a severe mental health problem and an illness, and by Perre et al. [34], where the length of time feeling depressed determined the difference between feeling down and being diagnosed with clinical depression. We further found that *depression* could be used in many situations, although it is not meant to be *real* clinical depression, and some participants underlined that it is important to use the word right; otherwise, the word will not be taken seriously. Hermann et al. [22] reported that adolescents’ thought peers commonly self-diagnosed depression without knowing what the condition entails (21–22).

However, there seems to be a lack of agreement on what strengths are most associated with mental health [38]. In his critical discussion on positive mental health, Vaillant [38] argues that positive mental health represents the presence of human strengths such as maturity, the dominance of positive emotions, high socioemotional intelligence, subjective well-being or resilience rather than the absence of weaknesses [38].

### Experience-based manifestations of being – an integration of two constructs of mental health?

Children and adolescents struggle to link their cognitive understanding of negative mental health concepts with their limited experiences with mental health issues. Moreover, they partly make use of these concepts when they describe experiences of feeling down or low. An approach to distinguish children and adolescents’ perceptions and articulations of mental health that has been suggested in several relevant studies is the social constructionist perspective on knowledge. A perspective of the history of psychiatry that demonstrates that the talk of *lighter mental symptoms* has changed over time. The analysis shows how the participants‘s descriptions of mental illness are characterized by the free use of psychological and psychiatric expert language and a *normalization* of different emotions, such as in contrast to the norms and demands of adult (expert) society [21, 39]. The elusiveness of the descriptions of being could very well relate to a constructivist merger of more advanced psychological expressions picked up by the adolescents from their immediate environment and from society at large with a self-reflective understanding of one’s feelings and moods. A perception of mental health that is similar to the dual-factor model [40]. A model that suggests a conceptualization of mental health consisting of two separate but still related constructs; an integration of subjective well-being with psychopathological symptoms [40]. The vague and trying expressions of being that our study recognized among the children could represent a sense of doubled awareness of either internalizing or externalizing mental disorders and well-being [41].

Worries were perceived as triggers of ill-being. Worries, manifested as stress about school and schoolwork, were also quite extensively expressed. It is clear that school seems to be the source of much anxiety and stress, which was confirmed in our interviews and elsewhere [3, 22]. In navigating expressions of positive and negative mental health in a school environment, it is interesting to note how Putwain et al. [42], in their network analysis, identified school-related well-being to be distinct from test anxiety and more serious conditions such as generalized anxiety disorder and panic disorder. Again, the dual-factor model suggests a way of separating but not disconnecting subjective expressions of well-being with psychological distress [40, 42].

Moreover, schools are the most common arena for promoting mental health and the most important because there is a possibility to reach children and adolescents who would not have been reached otherwise. An arena for promoting positive mental health but where stigma and negative attitudes are also addressed to encourage appropriate help-seeking for mental health problems. The latter represents more of a deficit approach although meta‐analyses show that multicomponent mental health promotion interventions are more effective when adopting a positive mental health stance rather than focusing exclusively on illness prevention [43].

Vague and elusive expressions of being presented by the children and adolescents should also be understood from the perspective of the professional. To what extent are we as researchers the cause of this vagueness? A question raised from the perspective that mental health has both a descriptive (*What is*) and a prescriptive (*What should be*) function. Manwell et al. [17] argue that the differences in how these functions are perceived must be empirically determined. In such an investigation, that we believe partly has been undertaken in this study, Huber et al. [16] emphasize that we must be aware of the risk of mixing scientific evidence with moral argument. A thought in line with the argument of the early criticisms of health-promoting perspectives, viewing health in positive, normative terms, avoiding a focus on risk and deficit [44]. The fact that children and adolescents struggle with sorting out the meaning of mental health might just reflect an academic uncertainty.

### Social norms – body image, mental health and social media links

Being normal and fitting in were considered essential to health. The results confirmed that it is important to look and act like everyone else. The concept of normality is ubiquitous in the debate on mental illness and mental health. Horowitz [45] argues that it is impossible to determine what constitutes a mental disorder without delineating what constitutes normal functioning. If everyday challenges increasingly are being medicalized and, as such, represent deviance from normality, this according to Aneshensel [46], raises the question of whether psychopathology constitutes a disjuncture with normality or is on the same continuum as normality, but at the opposite pole. However, Vaillant [38] contests the idea of the average being equal to healthy, meaning that at the community level the healthy individuals always mix with various forms of psychopathological presence [38]. The centrality and normativity of normality have been the subject of critical research [47]. From being an expression of the *probable*, it has become burdened with a discriminating power, therefore it has been scrutinized in medicine as well as in the fields of disability studies, gender studies, anthropology, psychiatry, cultural studies, sociology, and critical race studies [47].

Departing from these definitions, factors relating to the individual and one’s capacity and ability to interact with society can be identified, stressing concepts of agency, autonomy, and control. In the mapping by Manwell et al. [17] meaningful participation in valued roles such as family and work was recognized as important in understanding mental health. For children and adolescents, this would represent social role-taking in a school environment, but also social aspects such as being able to disconnect by choice, as opposed to being excluded. It is widely recognized that socially excluded adolescents are at increased risk of depression and anxiety [48]. The participants recognized a link between mental health, body image, and social media. The appearance was thought to influence one’s mental health, and this was exaggerated by Photoshopped influencers on social media. According to Choukas-Bradley et al. [49], social media constructs a “perfect storm” where idealized images of peers and quantifiable feedback, together with developmental factors and sociocultural gender socialization processes, amplify adolescent girls’ body image concerns. This can later lead to depression symptoms and disordered eating. The importance of social media for children and mental health cannot be emphasized enough, and adults need to raise awareness and be educated on this matter.

### Limitations

In this study, we interviewed a small sample of children; therefore, the results may not be directly transferable to other countries or age groups, although our findings are in line with previous research. Although the participants were recruited from different schools, we did not reach the most vulnerable areas; thus, the transferability to multicultural settings and rural areas is limited. Despite employing a nonclinical sample, several participants expressed personal interactions with counselors, doctors, and psychologists regarding mental health concerns, which may have influenced their views. We attempted to steer the discussion away from personal disclosures when these situations arose. Furthermore, the global COVID-19 pandemic and its associated restrictions and isolation for this target group may have affected their views on mental health. However, compared with other countries, Sweden faced fewer restrictions in closing compulsory schools, which might have positively influenced their mental health. Another consideration applies to the recruitment process; in some cases, the schools asked a few selected pupils if they wanted to participate rather than informing the whole class. More verbal and outwardly directed pupils were likely recruited over others. This might have influenced the richness and nuances of the interviews.

We used group interviews to encourage active discussions and enhance interactions by talking to one another, exchanging experiences, and commenting on each other’s points of view. However, the composition of the groups might have contributed to more or less interaction. For example, some participants might not have been comfortable talking in groups with the other sex, as the groups included both boys and girls. This could also be true for age differences within the group. There is also a risk of personal negative emotions toward other children in the group, influencing the dynamics of the discussions. Choosing girls and boys from different schools and of different ages may have enhanced the credibility of the data, as it might have offered a richer variation and understanding of mental health among children with different backgrounds. However, logistical constraints prevented this option from being feasible. We chose not to let the participants read and comment on the transcripts which could be considered a limitation [27], although we do not believe that this had a great impact on the analysis. Finally, we were two researchers at each interview, which could imply both pros and cons. It is possible that some children felt intimidated by having two adults ask them questions, but this was nothing we sensed was a problem.

## Conclusions

Health and mental health might be among the most important concerns for children and adolescents today. In an unstable and rapidly changing world where uncertainty dominates the global landscape, it is natural for individuals to feel worried. Transitioning from childhood to adolescence involves navigating various challenges, including friendships, social media, conformity to societal norms, and self-image, all of which significantly contribute to participants’ mental health, as articulated by them. The influence of social media and gender norms in adolescents’ lives has, according to the participants in our study, considerable effects on their mental well-being. It is imperative for adults to raise awareness and become educated on this matter. The participants’ familiarity with mental health concepts indicates a strong recognition of the terms, albeit not always fully comprehended. They demonstrated the ability to differentiate between mental health problems and everyday struggles more effectively than anticipated. However, how can we expect children’s understanding and expression of mental health and mental illness to match those that science might anticipate when several discourses and presumptions around the concept exist? When definitions and understandings of mental health have not remained stable, we, as researchers, have a great responsibility in finding that common language. Furthermore, it seems that children and adolescents are more aware of the negative side of mental health problems than the mental health-promoting side; thus, there is a need for more focus on positive mental health. We hope that our results will be used in schools by teachers, student health teams, and pupils in the ongoing health and mental health discussions and health promotion. Our results can hopefully support the process of developing a common language between adults and children. Future research should make use of this knowledge when creating health and mental health-promoting interventions and continue to ask children and adolescents about their views on their lives, and a specific focus should be on social media and how that affects their mental health.

## Electronic supplementary material

Below is the link to the electronic supplementary material.

Supplementary Material 1

## Data Availability

The collected data have been processed by personal data assistants. Audio files and printouts are stored at a secured server so that no unauthorized person has access to them. All information about the informants is encrypted and data will be saved for at least 10 years. Karlstad University is the responsible research principal and personal data controller. The university’s data protection officer could be reached at dpo@kau.se. To obtain access to the raw data analysed in our study, please contact Sven Hassler at sven.hassler@kau.se.

## Declarations

### Confirmation of manuscript not previously being published

The content of the manuscript has not been published, or submitted for publication elsewhere.

## Abbreviations

CAMHS: Child and Adolescent Psychiatry
WHO: The World Health Organization
SHS: Student Health Services
SH: Sven Hassler (Author)
SJS: Siri Jakobsson Støre (Author)
LP: Louise Persson (Author)
LB: Linda Beckman (Author)
COREQ: Consolidated Criteria for Reporting Qualitative Research
PsyD: Doctor of psychology
