# Supplementary material for: Children’s and adolescents’ views of health and mental health concepts - A qualitative group interview study
Source: BMC Public Health. 2024 Sep 16;24:2506. doi: 10.1186/s12889-024-20042-6 (PMC11403838; doi:10.1186/s12889-024-20042-6)
Supplement: Supplementary file 1 — Supplementary Material 1 [file 12889_2024_20042_MOESM1_ESM.docx]

SUPPLEMENTARY

INTERVIEW GUIDE FOR MIDDLE SCHOOL (grade 4 (10-11y) grade 5 (11-12y), grade 6 (12-13y))

What is "health" to you? Give examples of what health can be.

Is there "good" and "bad health"? Can you tell us about it/describe it?

How would you describe what "stress" is?

Have you heard of the word "mental health"? What do you think that means

What do you usually do to feel good?

If you feel sad, what do you usually do to feel good?

If you feel sad - who can you talk to?

If you feel worried - who can you talk to?

INTERVIEW GUIDE FOR HIGH SCHOOL (grade 7 (13-14y), grade 8(14-15y)

What does "health" mean to you? Can you describe it?

How would you describe "stress"?

How does one feel when feeling stressed?

How do you feel when you have to present in front of others at school?

What do you think of when you hear "mental health"? What does that mean to you?

If you feel sad/down, what do you usually do to feel good?

If you feel sad/down - who can you talk to?

Have you heard of words like "anxiety" and "panic disorder"? What do you think these words mean?

Some people are depressed. What do you think about depression? How are people who are depressed?

What would you like to learn more about health-related things at school? Why/Give examples.

If you are on social media, what do you usually do?

How do you experience social media? Is it a positive or negative environment?

Do you follow influencers? How do you experience these?

Some reports and research show that students today find school to be stressful. How do you feel about school?

Are there other things that can feel difficult to think about that have to do with the school environment or life in general?

What could the school do to make the students feel better during school hours?
